# Active aortic aneurysm sac treatment with shape memory polymer during endovascular aneurysm repair

**DOI:** 10.1016/j.jvscit.2023.101241

**Published:** 2023-06-12

**Authors:** Andrew Holden, Andrew A. Hill, Manar Khashram, Jan M.M. Heyligers, Arno M. Wiersema, Paul D. Hayes, Michel M.P.J. Reijnen

**Affiliations:** a Department of Radiology, Auckland City Hospital, Auckland, New Zealand; b Vascular Services, Auckland City Hospital, Auckland, New Zealand; c Department of Vascular and Endovascular Surgery, Waikato Hospital Hamilton, Hamilton, New Zealand; d Department of Surgery, Elisabeth-TweeSteden Ziekenhuis, Tilburg, The Netherlands; e Department of Surgery, Dijklander Ziekenhuis, Hoorn, The Netherlands; f St. John's Innovation Centre, Cambridge, United Kingdom; g Department of Surgery, Rijnstate, Arnhem, The Netherlands; h Multi-Modality Medical Imaging Group, University of Twente, Enschede, The Netherlands

**Keywords:** Abdominal aortic aneurysm, Aneurysm regression, Aortic endograft, Endovascular aneurysm repair, Shape memory polymer

## Abstract

Preprocedural image analysis and intraprocedural techniques to fully treat infrarenal abdominal aortic aneurysm sacs outside of the endograft with shape memory polymer (SMP) devices during endovascular aneurysm repair were developed. Prospective, multicenter, single-arm studies were performed. SMP is a porous, self-expanding polyurethane polymer material. Target lumen volumes (aortic flow lumen volume minus endograft volume) were estimated from the preprocedural imaging studies and endograft dimensions. SMP was delivered immediately after endograft deployment via a 6F sheath jailed in a bowed position in the sac. Technical success was achieved in all cases, defined as implanting enough fully expanded SMP volume to treat the actual target lumen volume.

Abdominal aortic aneurysm (AAA) sac regression after endovascular aneurysm repair (EVAR) is associated with improved survival compared with stable or expanding sacs.^1^ Preemptive coil or plug embolization of the inferior mesenteric artery and lumbar arteries has been shown to decrease the rate of persistent type II endoleaks.^2^ Nonselective coil embolization of the AAA sac at the same time as EVAR has shown greater sac regression and lower type II endoleak rates compared with nonembolized AAA sacs in randomized controlled trials.^3^^,^^4^ The embolic material volume might factor into the extent of sac regression.^3^^,^^5^ However, coils result in imaging artifacts that preclude imaging of endoleaks, and the procedure is difficult to standardize.

Shape memory polymer (SMP) is a novel radiolucent material that self-expands to a porous scaffold on delivery into a vessel and is designed to support thrombus formation throughout its structure. In animal studies, SMP formulations have been shown to stimulate the immune response without chronic inflammation, and SMP bioabsorbs over time.^6^ SMP devices have been used for vessel embolization indications.^7^, ^8^, ^9^, ^10^ The feasibility of active AAA sac management with SMP during EVAR has been evaluated.^11^ We describe the development of preprocedural analysis and intraprocedural techniques to maximize aortic lumen thrombosis with SMP immediately after endograft placement.

## Methods

Two equivalent prospective, multicenter, single-arm studies are ongoing in New Zealand (ClinicalTrials.gov identifier, NCT04227054) and the Netherlands (ClinicalTrials.gov identifier, NCT04751578). The Northern-A Health and Disability Ethics Committee and Commissie Mensgebonden Onderzoek Regio Arnhem-Nijmegen approved the studies (corresponding approval nos. 20/NTA/4 and 2021-7370). The participants were consecutive adult candidates for elective EVAR of an infrarenal AAA. The key exclusion criteria were an aortoiliac aneurysm that could not be adequately sealed in the proximal common iliac artery, patent feeding vessels >4 mm in diameter, and a target lumen volume of <20 mL or >135 mL. The Appendix (online only) contains a complete list of the eligibility criteria. Major adverse events were defined as all-cause mortality, bowel ischemia, myocardial infarction, paraplegia, renal failure, respiratory failure, stroke, and procedural blood loss >1000 mL. All included patients gave written informed consent before undergoing any study procedures. The studies have completed enrollment, and follow-up is ongoing. Longer term safety and 1-year efficacy outcomes will be reported separately. The data are subject to change according to findings from ongoing data monitoring.

The primary end point of the studies was technical success, defined as filling the actual target lumen volume with expanded SMP.

### Innovative technique

**Fig 1:**
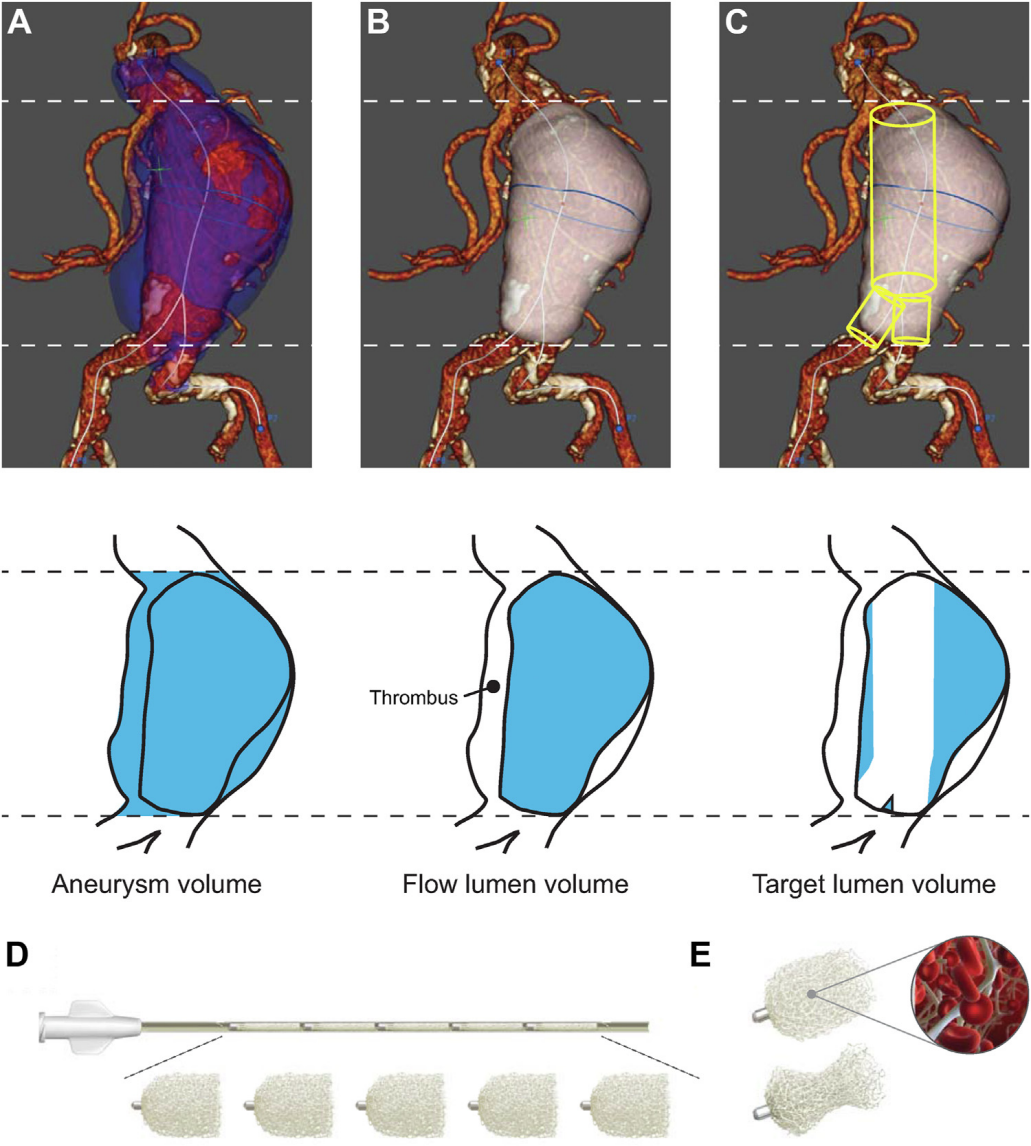
**A,** The aneurysm volume is the flow lumen volume plus any preexisting thrombus. **B,** The EndoSize analysis tool was used to determine the flow lumen volume, with manual confirmation of correct boundary identification. **C,** Cylinders based on the endograft diameters from the instructions for use and sac centerline lengths were used to estimate the endograft volume within the flow lumen volume. The estimated target lumen volume for treatment with shape memory polymer (SMP) is the flow lumen volume exclusive of the endograft volume. **D,** The contents of each IMPEDE-FX RapidFill device occupies ≤6.25 mL when the SMP is fully expanded. **E,** SMP is porous and has low radial force.

A total of 35 patients (30 men [86%]; mean age, 76 ±7.2 years) were treated from September 2020 through August 2022. The key preoperative aneurysm characteristics are summarized in the Table. Preprocedural computed tomography angiography obtained ≤3 months before the procedure was used to calculate the sac diameter and flow lumen volume and estimate the target lumen volume by deducting the estimated endograft volume from the flow lumen volume (Fig 1 and Supplementary Video, online only). Reference points just below the lowest renal artery and at the termini of the sac along each iliac artery were used for consistent volume estimates. The EndoSize volume analysis tool (Therenva, Rennes, France) was used to calculate the flow lumen volume, with manual confirmation of correct boundary identification. Sac centerline diameters were determined, corresponding to the widest point of the sac. Expanded endograft volumes were estimated from the instructions for use for the diameters and sac centerline lengths. The target lumen volume was the flow lumen volume exclusive of the endograft volume. The estimated target lumen volume was used to determine the number of IMPEDE-FX RapidFill devices (Shape Memory Medical) needed for each case (Table). The porous SMP in each device expands to occupy ≤6.25 mL.

**Table:** Preoperative aneurysm characteristics, endograft, and treatment details (n = 35)

Characteristic	Value
Aneurysm diameter, mm	61 ± 8.7
Aneurysm flow lumen volume, mL	108 ± 29
Endografts^a^	
Medtronic Endurant II/IIs	18 (51)
Gore Excluder	12 (34)
Gore Excluder Conformable	5 (14)
Target lumen volume, mL	56 ± 27
Approach	
Contralateral	14 (40)
Ipsilateral	21 (60)
Shape memory polymer devices^b^	10 (8-15)
Shape memory polymer volume/target lumen volume estimate ratio^c^	1.4 ± 0.3

Data presented as mean ± standard deviation for continuous variables or number (%) for categorical variables.

a Ninety-nine percent based on rounding.

b Number of IMPEDE-FX RapidFill devices (occupies ≤6.25 mL when shape memory polymer is fully expanded) presented as median (interquartile range).

c Maximum volume occupied by the implanted fully expanded shape memory polymer/estimated target lumen volume from preprocedural imaging analysis.

Bilateral femoral artery access was established per the endograft instructions for use. Either an ipsilateral or a contralateral approach was used (Supplementary Video, online only). Contralateral delivery of the SMP required upsizing the introducer by 2F. If the ipsilateral endograft limb landed above the aortic bifurcation, ipsilateral delivery was possible, which did not require access upsizing.

After deployment of the endograft main body, a hydrophilic guidewire and catheter were positioned around the outer circumference of the aneurysm sac blood lumen and were then replaced with a medium-support J-tip, 0.035-in. guidewire. The limb was deployed parallel to the guidewire, which was consequently jailed between the endograft and the artery wall. After both limbs were deployed, balloon dilatation of the endograft in the infrarenal neck region, overlap zone between the main body and limb, and distal seal zone not containing the jailed guidewire was performed. Angiography was used to confirm the absence of type I and III endoleaks.

**Fig 2:**
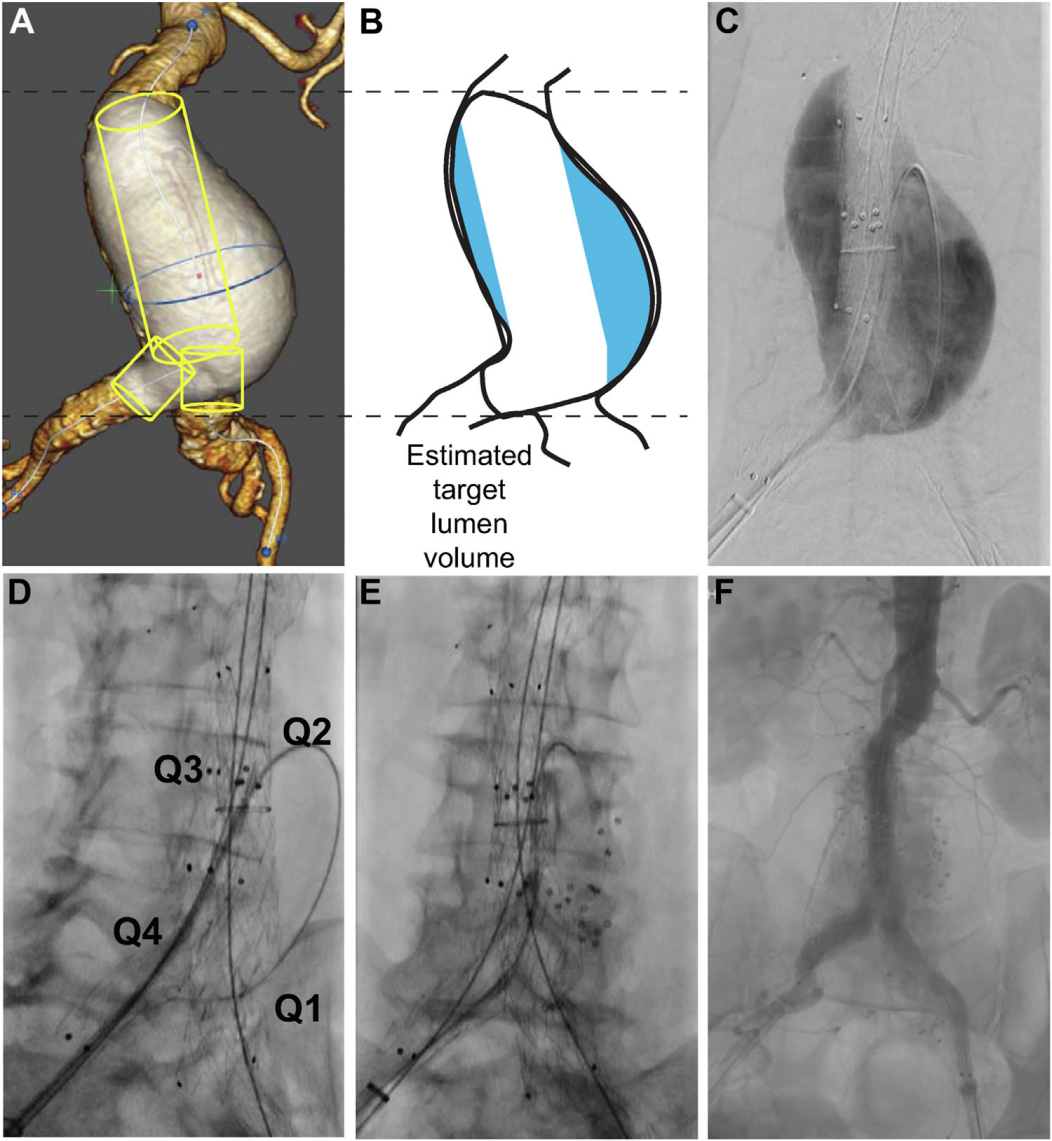
Deployment of shape memory polymer (SMP) throughout the actual target lumen volume of an abdominal aortic aneurysm (AAA) sac with an ipsilateral approach. **A,** The flow lumen volume (*white*) was 128 mL based on preprocedural imaging. **B,** The estimated target lumen volume was 86 mL (flow lumen volume minus the endograft volume). **C,** Intraprocedural sacogram showing the actual target lumen volume. **D,** A flexible 6F sheath was advanced over the jailed guidewire positioned around the circumference of the aneurysm sac blood lumen into the first caudal quadrant (*Q1*). The other quadrants are also labeled (*Q2-Q4*). **E,** After deployment of SMP into the first caudal quadrant, visible via small radiopaque markers. **F,** Case completion angiogram showing 94 mL of SMP distributed throughout the target lumen volume (based on the fully expanded volume of the devices implanted). The ratio of implanted SMP volume to the preprocedural estimate of the target lumen volume was 1.1, highlighting the role of intraprocedural monitoring in ensuring complete treatment of the actual target lumen volume.

A flexible 6F sheath (inner diameter, 0.070-0.090 in. to accommodate the SMP devices and minimize the potential of any friction as the SMP starts to expand on contact with blood) was advanced over the jailed guidewire, around the circumference of the blood lumen, to the first caudal quadrant (Fig 2 and Supplementary Video, online only). Imaging after manual contrast injection confirmed positioning and visualized the aortic branch vessel ostia. Using a 0.035-in. guidewire, ∼25% of the estimated volume of the SMP (ie, 25% of the estimated number of devices) was then slowly pushed and distributed into the quadrant, with a 5-minute pause for SMP expansion before moving to the next quadrant. The transition from dynamic to stationary radiopaque device markers signaled sufficient expansion. The sheath was then retracted circumferentially to deliver SMP into each of the cephalad quadrants (both left and right sides) and, finally, into the remaining caudal quadrant of the sac. Manual contrast injections were used to monitor treatment progress, ensure SMP was delivered near the branch vessel ostia, and confirm comprehensive sac treatment at case completion.^12^ After sheath removal, balloon dilatation sealed the working endograft limb.

### Procedural and perioperative (30-day) safety results

Complete technical success was achieved with treatment of the actual target lumen volume in all cases (Table). Fig 2, *F* shows an example of device distribution seen on case completion angiography.

No device or study procedure-related major adverse events occurred through 30 days. Two patients experienced fever, which was attributed to device-related postimplantation syndrome 1 to 3 days after the procedure. Both cases resolved without sequelae. The Supplementary Table includes other device- and procedure-related periprocedural serious adverse events.

## Discussion

The procedure descriptions we provide are a result of our progressive experience during the studies. The learning curve to establish the techniques to distribute SMP into and throughout infrarenal AAA sacs was relatively short and built on feasibility experience.^11^ The quadrant-based approach minimized sheath movement and disruption to the endograft within the sac with a single retraction path and proved to be effective in delivering SMP throughout the sac. With respect to room within the sac, our eligibility criteria limited the sac diameter to ≥55 mm for men and ≥50 mm for women, and 51-mm-diameter sacs were treated without issue using this technique. We believe the 5-minute pauses for SMP expansion before filling the next quadrant contributed to SMP distribution in the sac, especially near the branch vessel ostia. The preprocedural target lumen volume estimates facilitated case planning; however, the sacs were filled based on intraprocedural observations. An important feature of SMP with this approach is its low radial force.

We used flexible guiding sheaths to minimize the potential for aneurysm wall injury and endograft dislodgement, especially when positioning the sheath in the first quadrant. With this technique, the deployment of SMP devices was unremarkable in our experience. With experience and reflection, we believe a steerable sheath could facilitate positional control to fully treat quadrants in challenging anatomies or when targeting branch vessel ostia but should be used with caution to minimize the potential for sac perforation or endograft displacement during SMP delivery. Fibrin glue and thrombin/Gelfoam (Pfizer) have also been used to treat AAA sacs.^3^^,^^12^^,^^13^ SMP device treatment of AAA sacs offers the potential for controlled and predictable placement of a material that supports thrombus formation throughout the sac.

The goal of these studies was to establish the procedural steps for larger studies; however, a limitation is the relatively small sample size. Application of the technique to smaller sacs than those included also requires further study.

## Conclusions

Techniques to plan and execute procedures to treat infrarenal AAA sacs with SMP during EVAR were established. The target lumen volume of the aneurysm sacs outside of endografts were treated with SMP, based on the maximum volume of the expanded material. The 30-day safety profile of sac treatment was acceptable in this small safety study, and safety and efficacy evaluations are ongoing.

## Figures and Tables

**Supplementary Table:** Periprocedural (30-day) serious adverse events^a^

Serious adverse event	Device related^b^	Procedure related^b^	Time after procedure,^c^ days	Status^d^
Hyperglycemia	No	Yes	0	Resolved without sequelae
Arrhythmia	No	Yes	1	Resolved without sequelae
Postimplantation syndrome	Yes	No	1	Resolved without sequelae
Fever	Yes	No	1	Resolved without sequelae
Constipation	No	Yes	2	Resolved without sequelae
Low hemoglobin	No	Yes	2	Resolved with sequelae
Postimplantation syndrome	Yes	No	3	Resolved without sequelae
Abdominal pain	No	Yes	5	Resolved without sequelae

a Serious according to ISO 14155:2020 Clinical investigation of medical devices for human subjects – good clinical practice.

b Probably or more likely related to the study device or study procedure, which was adjudicated by a medical monitor independently of the study investigators.

c Day 0 is the day of procedure.

d Status at last follow-up.

## Appendix

*Additional material for this article may be found online at**www.jvscit.org*.

## APPENDIX

Supplementary Video (online only) Step-by-step examples of preprocedural planning, contralateral and ipsilateral approaches for the delivery sheath, and deployment of the shape memory polymer (SMP) devices throughout the actual target lumen volume of an abdominal aortic aneurysm (AAA) sac.

Study inclusion criteria:

1. Age ≥18 years
2. A candidate for elective endovascular aneurysm repair of an infrarenal aortic aneurysm ≥5.5 cm in diameter in men and ≥5.0 cm in women

Study exclusion criteria:

1. An inability to provide informed consent
2. Enrolled in another clinical study
3. Aortoiliac aneurysm or concomitant iliac artery ectasia or aneurysm (common iliac artery diameter >24 mm) close to the bifurcation and/or that could not be adequately sealed
4. Patent abdominal aortic aneurysm (AAA) sac feeding vessels (within the sac) >4 mm in diameter
5. Volume of AAA sac to be filled after stent graft placement <20 mL or >135 mL based on preprocedure computed tomography angiogram (ie, aortic flow volume exclusive of stent graft volume)
6. Use of aortic stent grafts other than the Gore Excluder AAA endoprosthesis, Cook Zenith Flex AAA endovascular graft, Medtronic Endurant II stent graft, or Endologix Ovation Alto abdominal stent graft system to treat the AAA
7. Planned use of the chosen stent graft outside its instructions for use
8. Planned use of fenestrated or chimney stent grafts
9. Study participants in whom stent graft placement is abandoned for any reason and/or for whom the investigator decides, during the course of stent graft placement, that the study procedure might not be appropriate
10. Planned use of embolic devices other than the investigational product to embolize the AAA sac
11. Vascular disease and/or anatomy that preclude safe access and positioning of a catheter to deliver the investigational product into the AAA sac
12. Ruptured, leaking, or mycotic (infected) aneurysm
13. Aneurysmal disease of the descending thoracic aorta
14. Coagulopathy or uncontrolled bleeding disorder
15. Long-term (>6 months before the procedure) use of a direct oral anticoagulant or any vitamin K antagonist anticoagulant use
16. Serum creatinine level >2.5 mg/dL
17. Cerebrovascular accident within 3 months before the procedure
18. Myocardial infarction and/or major heart surgery within 3 months before the procedure
19. Atrial fibrillation that is not well rate controlled
20. Unable or unwilling to comply with study follow-up requirements
21. Life expectancy of <2 years after procedure
22. Known hypersensitivity or contraindication to platinum, iridium, or polyurethane
23. A condition that inhibits radiographic visualization during the implantation procedure
24. History of allergy to contrast medium that cannot be managed medically
25. Uncontrolled comorbid medical condition, including mental health issues, that would adversely affect participation in the study
26. Pregnancy or lactation; for women of child-bearing potential, pregnancy was determined by a positive pregnancy test within 7 days before the procedure or refusal to use a medically accepted method of birth control for the duration of the study
27. For possible participants living in New Zealand: prisoner or member of another vulnerable population; for those living in the Netherlands: a member of a vulnerable population
